# Identification of MAN1B1 as a Novel Marker for Bladder Cancer and Its Relationship with Immune Cell Infiltration

**DOI:** 10.1155/2022/3387671

**Published:** 2022-08-16

**Authors:** Xueping Wang, Bin Chen, Yifang Cao, Yi He, Wei Chen

**Affiliations:** Department of Urology, The Affiliated Hospital of Jiaxing University, Jiaxing, Zhejiang, China

## Abstract

Bladder cancer (BC) is a common malignant tumor of the genitourinary system, and there are not enough tumor biomarker tests that are specific, trustworthy, and noninvasive for the diagnosis and prognosis. The purpose of this study is to investigate the clinical relevance, prognostic value, and immunological signature of Mannosidase alpha class 1B member 1(MAN1B1) expressions in BC. The Cancer Genome Atlas (TCGA) and Genotype-Tissue Expression (GTEx) databases provided the raw information that was used to analyze the expression of MAN1B1 in tumor patients. Then, a statistical study was carried out to assess the correlations of MAN1B1 expression with the clinical characteristics and the prognosis of BC. The correlation between MAN1B1 expression and tumor immune infiltration was explored via single-sample gene set enrichment analysis (ssGSEA). In human cancers, MAN1B1 expressions were shown to be generally higher in tumors than in normal specimens. We confirmed that MAN1B1 expression was distinctly increased in BC specimens compared with nontumor specimens. BC specimens with advanced T stage and M stage showed a higher level of MAN1B1. Survival analysis revealed that the overall survival (OS), disease-specific survival (DSS), and progression-free interval (PFI) of patients with high MAN1B1 expressions were distinctly worse than those with low MAN1B1 expressions. Importantly, multivariate analyses only confirmed that MAN1B1 expression was an independent prognostic factor for OS of the patients with BC. Furthermore, we observed that MAN1B1 expression level was significantly correlated with abundance of multiple immune infiltrates including Th2 cells, macrophages, Th1 cells, neutrophils, T helper cells, and NK CD56 bright cells. In conjunction with all of these findings, elevated MAN1B1 expression is associated with a poor prognosis and a higher number of immune cells in BC. MAN1B1 has the potential to act as a biomarker that can evaluate both the patient's prognosis and the degree of immune infiltration in BC.

## 1. Introduction

Bladder cancer (BC) is the fourth most prevalent cancer in men and the most frequently diagnosed malignancy of the urinary system worldwide [1]. The risk of developing BC increases with age, and there has been a discernible rising trend in the overall incidence of BC over the past several years [2, 3]. Approximately 90 percent of all cases of cancer are classified as transitional cell carcinoma. The majority of bladder cancers are known as nonmuscle-invasive bladder cancers (NMIBCs), which frequently recur and progress into muscle-invasive bladder cancers [4, 5]. MIBCs account for around 30 percent of all cases of BC, and the treatment of choice for these cases is radical cystectomy in conjunction with pelvic lymph node dissection [6, 7]. In contrast to the majority of other malignancies, the diagnostic procedures, treatment options, and percentage of patients who are still alive after five years have not altered in the past three decades [8, 9]. With a recurrence rate of between 60 and 70 percent, BC continues to be a significant risk to human health all over the world. As a consequence of this, it is necessary to create methods of precise prediction in order to advance clinical diagnosis and therapy.

Mannosidase alpha class 1B member 1(MAN1B1), located on 9q34.3, encodes an enzyme belonging to the glycosyl hydrolase 47 family [10]. This enzyme has a role in N-glycan biosynthesis and belongs to the class I alpha-1,2-mannosidase family [11]. It converts Man9GlcNAc to Man8GlcNAc isomer B in a very particular manner. MAN1B1 encoded *α*-1,2-mannosidase *α*-Mannosidase is known to have an important role in both the modification of protein glycosylation and the hydrolysis of glycoprotein polysaccharides [12, 13]. It is composed of many different classes, including -Mannosidases I, -Mannosidases II, and unclassified -Mannosidase [14]. Evidence indicating alpha-mannosidase has a role in the progression of cancer has been accumulating steadily over the years [15–17]. However, the potential function of MAN1B1 in tumors was rarely reported. In the current investigation, our goals were to investigate the predictive significance of MAN1B1 in BC and to investigate its connection with immune infiltration.

## 2. Materials and Methods

### 2.1. Raw Data Acquisition and Processing

The Cancer Genome Atlas (TCGA) research network has profiled and evaluated a massive collection of clinical data on > 10,000 cancer patients representing 33 distinct types of tumors. Downloading clinical data and RNA expression information from the TCGA and Genotype-Tissue Expression (GTEx) databases was accomplished through the usage of the UCSC Xena database (https://xenabrowser.net/datapages/). 33 cancer types were included: OV, PAAD, PRAD, READ, SKCM, STAD, TGCT, THCA, THYM, UCEC, UCS, ACC, BLCA, BRCA, COAD, DLBC, ESCA, GBM, HNSC, KICH, KIRC, KIRP, LAML, LGG, LIHC, LUAD, and LUSC.

### 2.2. Analysis of MAN1B1 Expression in Cancers

The TCGA and GTEx projects were the sources for the information regarding the abnormal expressions of MAN1B1 between cancer and normal specimens that were matched to the tumor. A tissue bank and data resource known as GTEx (https://gtexportal.org) was established by the National Institutes of Health (NIH) Common Fund. In total, 53 human normal specimens from approximately 1,000 individuals have been analyzed for RNA sequencing, genetic variation and molecular phenotypes. Plotting was done using log2 (TPM + 1) converted expression data, which was our choice for the parameter selection process.

### 2.3. Cox Regression Analysis and Survival Analysis

In order to investigate the connection between MAN1B1 expressions and patients' overall survival (OS), disease-specific survival (DSS), and progression-free interval (PFI) in bladder urothelial carcinoma(BLCA) patients, a Cox regression analysis was carried out in the R environment. After using the best approach for separating patients into groups with high and low MAN1B1 expression, the Kaplan–Meier methods were applied to produce survival curves for patients. This was done after sorting patients into groups with high or low MAN1B1 expressions. An examination of the survival was carried out with the aid of survival receiver operating characteristics (ROC) and the survival package in R. The log-rank test was applied to analyze the differences between the curves, and a *p* < 0.05 was considered statistically significant.

### 2.4. Immune Cell Infiltration

Using ssGSEA, the relative degrees of tumor infiltration by 24 different immune cell types were assessed. This allowed us to investigate the expression of genes found in published signature gene lists. The signatures that we used contained a total of 509 genes and included a wide variety of cell types involved in both adaptive and innate immune responses. The Wilcoxon rank sum test and Spearman correlation were utilized in order to investigate whether or not there is a connection between MAN1B1 and the infiltration levels of immune cells, as well as whether or not there is an association between the infiltration levels of immune cells and the various expression groups of MAN1B1.

### 2.5. Statistical Analysis

All statistical analyses were performed in the R package (V3.6.2). The Wilcoxon test was utilized in order to compare the differences between two groups of data that were not regularly distributed. The relationship between clinical pathologic features and MAN1B1 was analyzed with the Wilcoxon signed-rank sum test and logistic regression. ROC curves were established to evaluate the diagnostic value of MAN1B1 in BC patients. A *p* < 0.05 was considered statistically significant.

## 3. Results

### 3.1. MAN1B1 Expression in Pan-Cancer

**Figure 1:**
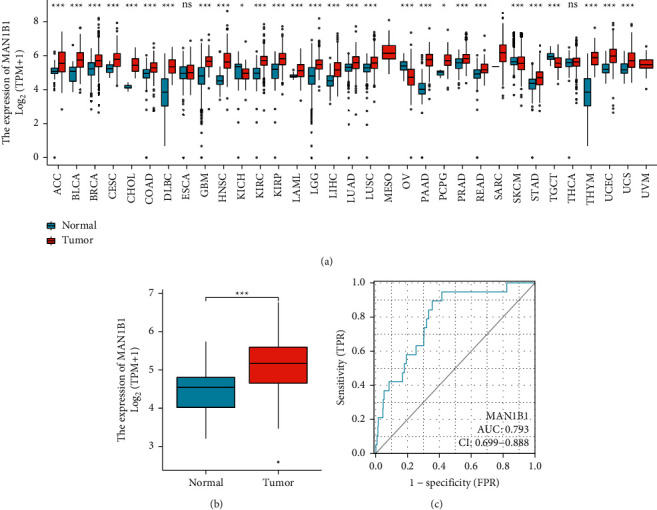
TCGA and GTEx databases show MAN1B1 expression in both healthy and cancerous tissues. (a) pan-cancer analysis. (b) MAN1B1 expression was increased in BC specimens compared with nontumor specimens. (c) MAN1B1's ROC in BC. on the *X*-axis, false-positive rates are measured; on the *Y*-axis, true-positive rates are measured. ^*∗*^*p* < 0.05, ^*∗∗*^*p* < 0.01, ^*∗∗∗*^*p* < 0.001.

**Figure 2:**
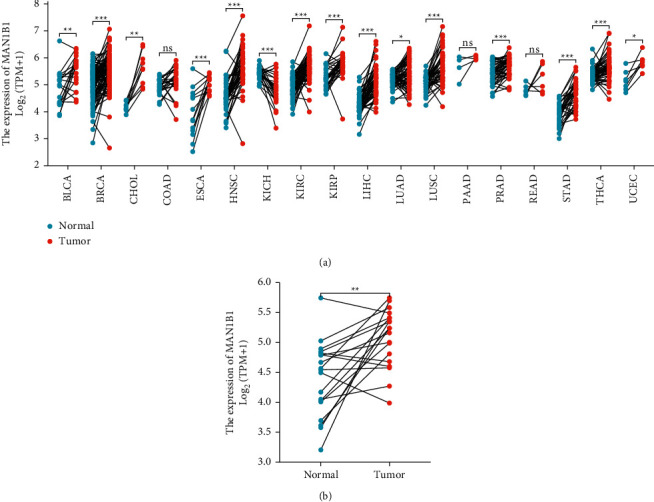
TCGA and GTEx databases' unpaired *t*-tests reveal MAN1B1 expression in healthy and cancerous tissues. (a) pan-cancer analysis. (b) MAN1B1 expression in BC and nontumor specimens. ^*∗*^*p* < 0.05, ^*∗∗*^*p* < 0.01, ^*∗∗∗*^*p* < 0.001.

Based on TCGA datasets, we investigate the level of MAN1B1 expression in each of the many cancer types.  Figure 1(a) showed that the levels of MAN1B1 expression in the tumor specimens of ACC, BLCA, BRCA, CESC, CHOL, COAD, DLBC, GBM, HNSC, KICH, KIRC, KIRP, LAML, LGG, LIHC, LUAD, LUSC, SARC, STAD, THYM, UCEC, and UCS are all higher than those in the nontumor tissues. However, the expression of MAN1B1 in the tumor tissues of SKCM and TGCT was significantly lower than that in the normal tissues (Figure 1(a)). The expression of MAN1B1 in BLCA is shown in Figure 1(b). Due to the considerable overexpression of MAN1B1 in BLCA patients, we were motivated to further investigate the diagnostic significance of this gene for BLCA patients. As shown in Figure 1(c), the results of ROC curves revealed that the AUC was 0.793 (0.6999 to 0.888, 95% CI) in a comparison between BLCA specimens and nontumor specimens. In addition, we also performed a paired *t*-test. A similar finding was also observed (Figures 2(a) and 2(b)). Our findings suggest that overexpression of MAN1B1 in malignancies may be a common occurrence.

### 3.2. Association between Clinicopathological Characteristics and MAN1B1 Expression in BLCA Patients

**Figure 3:**
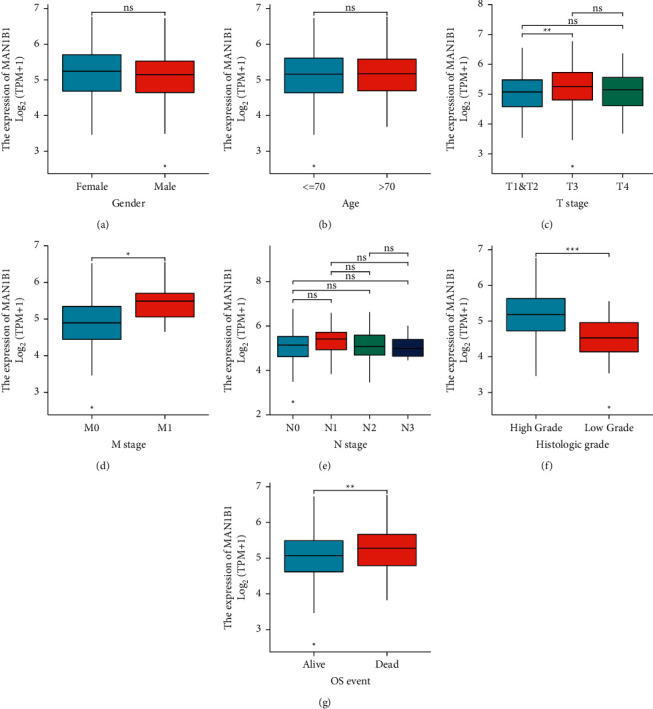
The associations between MAN1B1 expressions and several clinical factors. (a) gender, (b) age, (c) T stage, (d) M stage, (e) N Stage, (f) histologic grade, (g) OS event. ^*∗*^*p* < 0.05, ^*∗∗*^*p* < 0.01, ^*∗∗∗*^*p* < 0.001.

We evaluated the association between the expression of MAN1B1 and the clinicopathological aspects of BLCA in order to further define the relevance of MAN1B1 in BLCA. This was done in order to better understand the role that MAN1B1 plays in BLCA. On the basis of the median relative MAN1B1 expression value, all of the patients diagnosed with BLCA were separated into two groups. As shown in Table 1, we did not observe a distinct association between MAN1B1 expression and several clinical factors based on the results of the chi square test. In the *T*-test, we also did not observe a distinct association between MAN1B1 expression and gender and age (Figures 3(a) and 3(b)). However, we found that BLCA specimens with advanced T stage and M stage showed a higher level of MAN1B1 (Figures 3(c) and 3(d)). The expression of MAN1B1 did not change distinctly in specimens with different N stages (Figure 3(e)). Moreover, we found that the expression of MAN1B1 was distinctly increased in BLCA specimens with high grade or dead status (Figure 3(f) and 3(g)).

### 3.3. Associations between MAN1B1 Expression and Patient Survival

**Figure 4:**
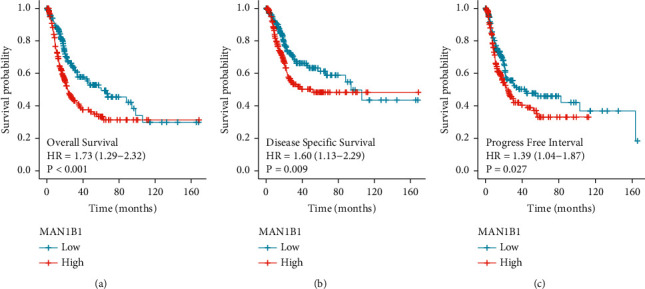
Kaplan–Meier curves estimating the OS, DSS, and PFI rates according to the expressions of MAN1B1 in patients with BC.

To explore the relationships between MAN1B1 expressions and survivals of BLCA patients, Kaplan–Meier methods were performed to analyze the differences in OS, DSS, and PFI. As shown in Figure 4, the OS, DSS, and PFI of patients with high MAN1B1 expressions were distinctly worse than those with low MAN1B1 expressions. In univariate analyses, we observed that MAN1B1 expression was associated with OS (Table 2), DSS (Table 3), and PFI (Table 4) of BLCA patients. However, multivariate analyses only confirmed that MAN1B1 expression (HR = 1.970, 95% CI 1.226–3.167, *p*=0.005) was an independent prognostic factor for OS of the patients with BLCA (Table 2). The potential prognostic values of MAN1B1 used as an independent prognostic factor for DSS and PFI of BLCA patients were not further confirmed (Tables 3 and 4).

### 3.4. The Correlation between MAN1B1 Expression and Immune Infiltration

**Figure 5:**
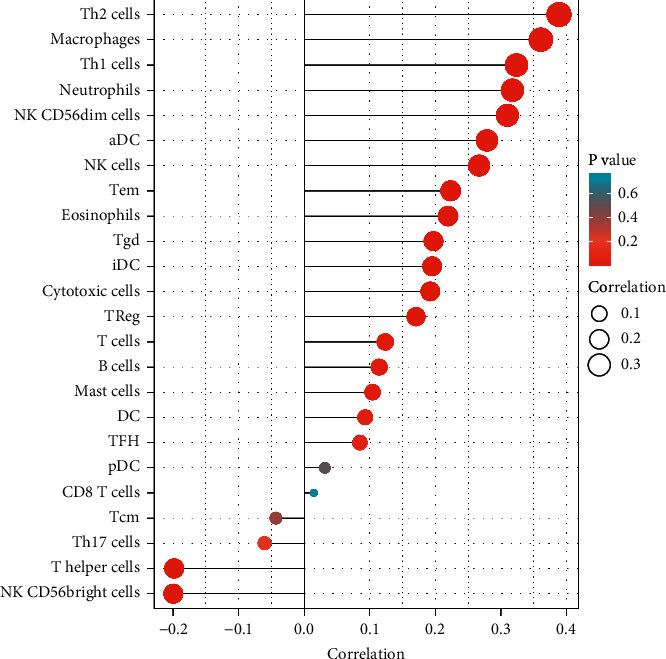
Lollipop chart of MAN1B1 expressions in 24 immune cells. The levels of MAN1B1 were associated with the levels of several immune cells.

The spearman correlation test was used to investigate the relationship between the expression of MAN1B1 and the amount of immune cell infiltration that was measured using ssGSEA. The abundance of acquired immunocytes, such as T helper cells and NK CD56bright cells, was found to have a negative correlation with the expression of MAN1B1, while the abundance of innate immunocytes, such as Th2 cells, Macrophages, Th1 cells, Neutrophils, NK CD56dim cells, and other such cells, had a positive correlation with the expression of MAN1B1 (Figure 5).

## 4. Discussion

A number of cancers, including carcinomas, share a phenomenon known as deregulation of tumor-related genes, which plays a significant part in the progression of cancer via a variety of intricate pathways [18, 19]. Alterations in the expression of a particular gene have been shown to have a strong correlation both with the development of human malignancies and their overall prognosis [20, 21]. Thus, to better understand how BC develops and progresses, finding mRNA molecular profiles related to a patient's prognosis could reveal biological mechanisms at work. It could also lead to the discovery of new therapeutic targets for the disease.

MAN1B1 is a newly identified tumor-related gene. Previous study by Wang et al. reported that the expressions of MAN1B1 were distinctly higher in cancerous specimens than in nontumor samples. In addition, there was a correlation between increased MAN1B1 expression and a bad prognosis in patients with BC. In functional experiments, the suppression of MAN1B1 resulted in a reduction of BC cell proliferation, invasion, and migration [22]. To our best knowledge, this is the only research about the function of MAN1B1 in tumor. In this study, we first performed a pan-cancer analysis and observed that MAN1B1 expression was distinctly increased in most types of tumors, which was consistent with previous findings in BC. Our data revealed that MAN1B1 may serve as an oncogene in human tumors. Then, we further explored the relationships between MAN1B1 expressions and the prognosis of BC patients. We found that BC specimens with advanced stages showed an increased level. Interestingly, we found that the BC specimen with a dead event also showed an increased level of MAN1B1, suggesting that MAN1B1 may influence the clinical outcome of BC patients. The results of Kaplan–Meier methods confirmed that the OS, DSS, and PFI of patients with high MAN1B1 expression was significantly worse than those with low MAN1B1 expression. More importantly, multivariate analyses only confirmed that MAN1B1 expression were an independent prognostic factor for OS of the patients with BC. Our findings suggest MAN1B1 as a novel diagnostic and prognostic biomarker for BC patients.

Recent research studies have demonstrated that immune cells that infiltrate tumors, known as tumor infiltrating cells (TIICs), may be able to control the process of both the genesis and progression of tumors [23, 24]. In addition to this, TIICs have the capacity to undergo clonal expansion and are enriched preferentially in BC; a poor prognosis has been shown to correspond with an accumulation of TIICs in BC [25]. Then, our results demonstrated that MAN1B1 expression in BC was negatively associated with multiple types of immune cell infiltration. In previous studies, although it has been reported that the functions and prognostic relevance of numerous subtypes of TIICs in multiple forms of cancer are inconsistent, some of the results are still unclear. For example, infiltrating CD8+ T cells are generally considered to be tumor inhibitors that are associated with a positive prognosis in most types of cancer; however, in renal cell carcinoma and prostate cancer, CD8+ T cells are reported to be associated with poor clinical outcomes [26–29]. This is because infiltrating CD8+ T cells are associated with an increased risk of death. In addition, Hald et al. observed that CD8+ T cells were a predictor of a favorable prognosis in non-small-cell lung cancer [30]; however, the findings of Tian et al., who found that CD8+ T cells serve as a predictor of a less favorable prognosis, were in direct opposition to Hald et al. [31]. In macrophages, natural killer (NK) cells, and dendritic cells, researchers have obtained results that are similarly inconsistent. Due to the specialized roles and prognostic value of TIICs, additional samples and research work that are thoroughly developed are required to confirm the prognostic significance of these cells. In this investigation, we showed that the expression of MAN1B1 was connected with the presence of immune cells in the majority of tumors, which suggests that it may have an effect on the immunological status in BC.

There were several limitations to this study. First, since all of the data used in this study were obtained by downloading them directly from public sources and analyzing them using bioinformatics approaches, the results need to be validated using additional experimental research. Secondly, in order to investigate the possible connection between MAN1B1 and the cancer-related immune microenvironment in BC, more investigations were needed.

## 5. Conclusion

We discovered that MAN1B1 was overexpressed in BC and that its overexpression was significantly connected to a poor prognosis. There was a possibility that MAN1B1 plays a role in the progression of tumors and metastasis. In addition, our results suggest that MAN1B1 probably plays an important part in the polarization of macrophages and the infiltration of immune cells. Thus, it has the potential to be exploited as a prognostic target for BC.

## Figures and Tables

**Table 1:** Correlation between MAN1B1 expression with clinicopathologic features of BLCA.

Characteristic	Low expression of MAN1B1	High expression of MAN1B1	*p*
*n*	207	207	

T stage, *n* (%)			0.121
T1	4 (1.1%)	1 (0.3%)	
T2	65 (17.1%)	54 (14.2%)	
T3	86 (22.6%)	110 (28.9%)	
T4	32 (8.4%)	28 (7.4%)	

N stage, *n* (%)			0.111
N0	126 (34.1%)	113 (30.5%)	
N1	16 (4.3%)	30 (8.1%)	
N2	42 (11.4%)	35 (9.5%)	
N3	5 (1.4%)	3 (0.8%)	

M stage, *n* (%)			0.336
M0	129 (60.6%)	73 (34.3%)	
M1	5 (2.3%)	6 (2.8%)	

Gender, *n* (%)			0.655
Female	52 (12.6%)	57 (13.8%)	
Male	155 (37.4%)	150 (36.2%)	

Age, *n* (%)			0.921
<=70	118 (28.5%)	116 (28%)	
>70	89 (21.5%)	91 (22%)	

Age, median (IQR)	69 (60, 76)	68 (60, 76)	0.880

**Table 2:** Univariate and multivariate analysis of OS in BLCA patients.

Characteristics	Total (*N*)	Univariate analysis		Multivariate analysis	
		Hazard ratio (95% CI)	*p* value	Hazard ratio (95% CI)	*p* value
T stage	379				
T1 & T2	124	Reference			
T3	195	1.997 (1.358–2.939)	**<0.001**	1.975 (1.019–3.826)	**0.044**
T4	60	3.095 (1.934–4.954)	**<0.001**	2.572 (1.154–5.732)	**0.021**

N stage	369				
N0	239	Reference			
N1	46	1.858 (1.199–2.879)	**0.006**	1.634 (0.850–3.142)	0.141
N2 & N3	84	2.581 (1.828–3.646)	**<0.001**	2.176 (1.206–3.928)	**0.010**

M stage	213				
M0	202	Reference			
M1	11	3.136 (1.503–6.544)	**0.002**	1.028 (0.381–2.777)	0.956

MAN1B1	413				
Low	207	Reference			
High	206	1.728 (1.286–2.323)	**<0.001**	1.970 (1.226–3.167)	**0.005**

Age	413				
<=70	233	Reference			
>70	180	1.421 (1.063–1.901)	**0.018**	1.292 (0.804–2.075)	0.289

Gender	413				
Female	109	Reference			
Male	304	0.849 (0.616–1.169)	0.316		

**Table 3:** Univariate and multivariate analysis of DSS in BLCA patients.

Characteristics	Total (*N*)	Univariate analysis		Multivariate analysis	
		Hazard ratio (95% CI)	*p* value	Hazard ratio (95% CI)	*p* value
T stage	366				
T1 & T2	124	Reference			
T3	184	2.015 (1.262–3.216)	**0.003**	2.058 (0.873–4.853)	0.099
T4	58	3.243 (1.850–5.687)	**<0.001**	2.385 (0.851–6.682)	0.098

N stage	357				
N0	233	Reference			
N1	45	2.775 (1.675–4.599)	**<0.001**	2.259 (1.031–4.950)	**0.042**
N2 & N3	79	3.573 (2.341–5.452)	**<0.001**	2.896 (1.396–6.004)	**0.004**

M stage	208				
M0	197	Reference			
M1	11	4.205 (1.889–9.359)	**<0.001**	1.051 (0.337–3.282)	0.931

MAN1B1	399				
Low	204	Reference			
High	195	1.605 (1.126–2.286)	**0.009**	1.678 (0.915–3.076)	0.094

Age	399				
<=70	226	Reference			
>70	173	1.026 (0.718–1.466)	0.888		

Gender	399				
Female	103	Reference			
Male	296	0.849 (0.576–1.251)	0.408		

**Table 4:** Univariate and multivariate analysis of PFI in BLCA patients.

Characteristics	Total (*N*)	Univariate analysis		Multivariate analysis	
		Hazard ratio (95% CI)	*p* value	Hazard ratio (95% CI)	*p* value
T stage	380				
T1 & T2	124	Reference			
T3	196	1.848 (1.261–2.710)	**0.002**	2.354 (1.159–4.780)	**0.018**
T4	60	3.268 (2.043–5.227)	**<0.001**	2.934 (1.255–6.863)	**0.013**

N stage	370				
N0	239	Reference			
N1	46	2.394 (1.548–3.702)	**<0.001**	1.612 (0.824–3.155)	0.163
N2 & N3	85	3.157 (2.212–4.505)	**<0.001**	2.340 (1.274–4.300)	**0.006**

M stage	213				
M0	202	Reference			
M1	11	6.455 (3.117–13.367)	**<0.001**	1.770 (0.642–4.882)	0.270

MAN1B1	414				
Low	207	Reference			
High	207	1.393 (1.038–1.870)	**0.027**	1.478 (0.893–2.446)	0.128

Age	414				
<=70	234	Reference			
>70	180	1.066 (0.792–1.435)	0.673		

Gender	414				
Female	109	Reference			
Male	305	0.891 (0.642–1.235)	0.488		

## Data Availability

The datasets used and/or analyzed during the current study are available from the corresponding author upon reasonable request.
